# Uptake of early infant diagnosis in Thailand’s national program for preventing mother-to-child HIV transmission and linkage to care, 2008–2011

**DOI:** 10.7448/IAS.19.1.20511

**Published:** 2016-03-09

**Authors:** Thananda Naiwatanakul, Nipunporn Voramongkol, Niramon Punsuwan, Rangsima Lolekha, Robert Gass, Hansa Thaisri, Pranee Leechanachai, Mitchell Wolfe, Sarawut Boonsuk, Sorakij Bhakeecheep

**Affiliations:** 1 Division of Global HIV/AIDS and TB, Asia Regional Program, U.S. Centers for Disease Control and Prevention, Nonthaburi, Thailand; 2 Bureau of Health Promotion, Department of Health, Ministry of Public Health, Nonthaburi, Thailand; 3 Bureau of Epidemiology, Department of Disease Control, Ministry of Public Health, Nonthaburi, Thailand; 4 United Nations Children's Fund (UNICEF) Thailand, HIV/AIDS Section, Bangkok, Thailand; 5 Clinical Research Center, Department of Medical Science, Ministry of Public Health, Nonthaburi, Thailand; 6 Faculty of Medical Technology, Chiang Mai University, Chiang Mai, Thailand; 7 Division of Global HIV/AIDS and TB, Asia Regional Program, U.S. Centers for Disease Control and Prevention Atlanta, GA, USA; 8 The Thailand National Health Security Office (NHSO), Nonthaburi, Thailand

**Keywords:** EID, prevention of mother-to-child HIV transmission, Thailand, national PMTCT programme evaluation, linkage to care, antiretroviral therapy, HIV

## Abstract

**Introduction:**

Early infant diagnosis (EID) has been a component of Thailand's prevention of mother-to-child HIV transmission (PMTCT) programme since 2007. This study assessed the uptake, EID coverage, proportion of HIV-exposed infants receiving a definitive HIV diagnosis, mother-to-child transmission (MTCT) rates and linkage to HIV care and treatment.

**Methods:**

Infant polymerase chain reaction (PCR) testing data from the National AIDS Program database were analyzed. EID coverage was calculated as the percentage of number of HIV-exposed infants receiving ≥1 HIV PCR test divided by the number of HIV-exposed infants estimated from HIV prevalence and live-birth registry data. Definitive HIV diagnosis was defined as having two concordant PCR results. MTCT rates were calculated based on infants tested with PCR and applied as a best-case scenario, and a sensitivity analysis was used to adjust these rates in average and worst scenarios. We defined linkage to HIV care as infants with at least one PCR-positive test who were registered with Thailand's National AIDS Program. Chi-squared tests for linear trend were used to analyze changes in programme coverage.

**Results:**

For 2008 to 2011, the average EID coverage rate increased from 54 to 76% (*p*<0.001), with 65% coverage (13,761/21,099) overall. The number of hospitals submitting EID samples increased from 458 to 645, and the percentage of community hospitals submitting samples increased from 75 to 78% (*p*=0.044). A definitive HIV diagnosis was made for 10,854 (79%) infants during this period. The adjusted MTCT rates had significantly decreasing trends in all scenarios. Overall, an estimated 53% (429/804) of HIV-infected infants were identified through the EID programme, and 80% (341/429) of infants testing positive were linked to care. The overall rate of antiretroviral treatment (ART) initiation within one year of age was 37% (157/429), with an increasing trend from 28 to 52% (*p*<0.001).

**Conclusions:**

EID coverage increased and MTCT rates decreased during 2008 to 2011; however, about half of HIV-infected infants still did not receive EID. Most HIV-infected infants were linked to care but less than half initiated ART within one year of age. Active follow-up of HIV-exposed infants to increase early detection of HIV infection and early initiation of ART should be more widely implemented.

## Introduction

In 2014, an estimated 150,000 children under 15 years of age died of HIV-related causes worldwide [1]. HIV infection can be especially lethal for young children; mortality approaches 35% in the first year of life and 53% by two years, if untreated [2]. To reduce infant morbidity and mortality, the recently released WHO policy brief recommends antiretroviral treatment (ART) initiation for all HIV-infected infants and children [3], while the 2010 and 2014 Thai HIV treatment guidelines recommend immediate ART only in all HIV-infected infants less than one year of age regardless of CD4 count [4,5]. Early infant diagnosis (EID) of HIV may benefit infants and their families by promoting early infant access to HIV treatment, improving infant health, reducing early mortality [6–8], providing opportunities to link HIV-positive mothers and other family members with HIV to care during infant follow-up visits, and by providing reassurance to families of uninfected infants [9]. At the programme level, EID can help determine the effectiveness of prevention of mother-to-child transmission (PMTCT) programmes and support perinatal HIV elimination efforts [10].

Thailand's national guidelines for PMTCT of HIV recommend HIV counselling and testing for all pregnant women, initiating triple highly active antiretroviral therapy (HAART) for all HIV-positive pregnant women regardless of CD4 count (WHO Option B from 2010 [4] and WHO Option B plus from 2014 [5]), infant antiretroviral (ARV) prophylaxis for four weeks, counselling to encourage mothers to refrain from breastfeeding with the substitution of formula feeding for 18 months for all HIV-exposed infants and EID with HIV DNA polymerase chain reaction (PCR) at one to two months and four to six months. For infants with a positive PCR test result, guidelines recommend that a confirmatory PCR test be performed as soon as possible and that all HIV-exposed children also receive HIV antibody testing at 18 months of age regardless of PCR test results [4]. A national survey conducted in 2008 revealed that only 68% of infants born to HIV-positive mothers were tested at least one time for HIV and only 56% received confirmation of HIV diagnosis [11]. Data about linkage of HIV-infected infants to HIV care and treatment are not available.

**Figure 1:**
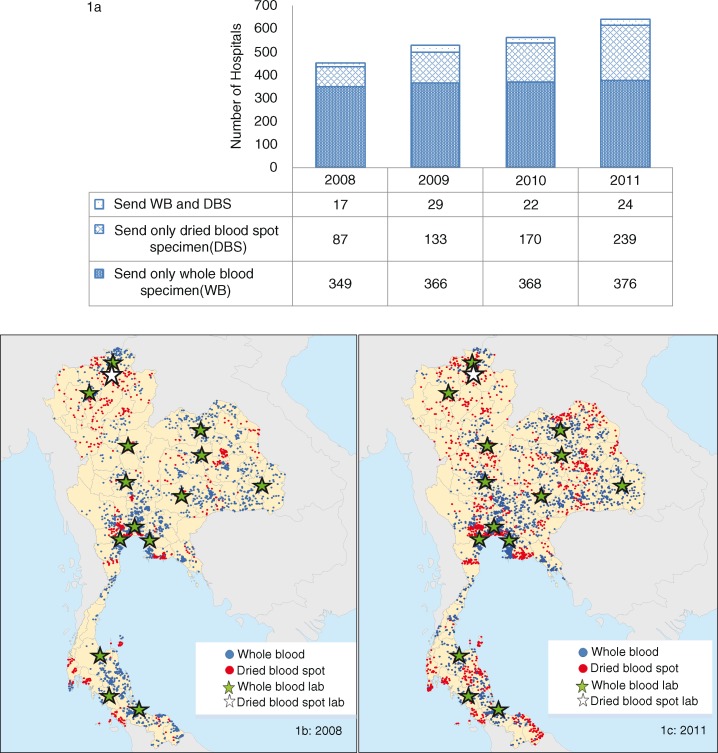
EID access and facility scale of sample collection. (a) Number of hospitals submitting EID samples by year and by type of specimen collection. (b and c) hospitals and location submitting EID samples in 2008 and 2011, respectively. Whole blood samples are collected in EDTA tubes, which can be transported by road to one of 14 Department of Medical Science (DMSc) laboratory networks (green star) within 48 hours and dried blood spot samples are collected on filter paper that can be sent via regular postal mail to Chiang Mai University Laboratory (white star). Blue dots are hospitals submitting whole blood samples and red dots are dried blood spot samples.

Implementing EID programmes is challenging because of the cost and complexity of collecting, transporting and testing biological samples and returning test results to providers and caregivers in a timely manner. EID services are currently available free of charge for all Thai infants (age <12 months old) in all public and private hospitals under Thailand's Universal Health Coverage (UC) Program (906 public health hospitals and 45 private hospitals). The 15 laboratory networks that perform PCR testing are geographically distributed in order to service the entire country (Figure 1b and c). A standardized and low-cost in-house conventional PCR has been optimized for the predominant HIV strains circulating in Thailand (CRF01_AE) using whole blood (WB) samples and dried blood spot (DBS) samples [12–14].

This study evaluated the Thailand National EID Program by examining changes in EID uptake and coverage over time, as well as changes in the proportion of HIV-exposed infants receiving a definitive HIV diagnosis and HIV-infected infants being linked to care and treatment services. This study also reports on mother-to-child transmission (MTCT) rates among PCR-tested infants and adjusted MTCT rates using sensitivity analysis, and compares trends during Thailand's use of different PMTCT regimens (initially WHO Option A, which changed to WHO Option B in 2010).

## Methods

This was a retrospective cohort study. We used the Thailand National AIDS Program (NAP) database to identify infants who received PCR testing between 2008 and 2011. Registration in the NAP database allows HIV-infected children to receive free, lifelong HIV treatment and care consistent with national HIV treatment guidelines in hospitals under the UC Program.

### Data collection

An online, centralized NAP database is maintained by the Thai National Health Security Office (NHSO) and used for managing national funds for HIV prevention, treatment and care. Information about each person who receives HIV treatment and care services is recorded in the database, and each person is given a unique national identification number (NID). Hospital staff enter data about infants (aged 0 to 12 months) born to (or suspected to have been born to) HIV-positive mothers in UC Program hospitals when a PCR test is requested. Test results are entered by laboratory staff who perform the HIV DNA PCR tests in the 15 laboratory networks. If infants or children receive a diagnosis of HIV infection, either by PCR or by HIV antibody test, they are registered in the NAP database and a unique NAP number is assigned, enabling them to receive HIV treatment and care in the UC system.

Study data related to EID, linkage to HIV care, ART initiation and CD4 count testing dates and results were abstracted from the NAP database using encrypted NAP and NID numbers. HIV-exposed infant data (month and year of birth, NID, date of HIV PCR testing request and facility that sent samples) were linked with HIV PCR test results. To determine national EID programme coverage, the number of HIV-exposed infants born each year was estimated using estimates of annual HIV prevalence among women delivering with and without antenatal care, as reported by the Ministry of Public Health's National PMTCT Monitoring System [15]. The number of annual live births was retrieved from the Birth Registration System maintained by the Ministry of the Interior's Bureau of Registration Administration [16].

### Definitions

EID uptake was defined as the number of HIV-exposed infants who received at least one PCR test in the NAP database. EID coverage was defined as the number of HIV-exposed infants that received at least one PCR test in the NAP database divided by the estimated number of HIV-exposed infants born in the same year. Definitive HIV infection was defined as two concordant positive PCR test results and noninfection as two concordant negative PCR test results. Presumptive HIV infection was defined as one positive PCR test result and presumptive noninfection as one negative PCR test result. Infants who had a negative first PCR test result and a positive second test result were included in the presumptive HIV-infected category. Infants who had only one negative PCR test result at age less than 30 days or who had conflicting PCR results (i.e. positive and then negative or indeterminate) were defined as inconclusive and excluded from the MTCT rate analysis.

Linkage to care was defined as infants with at least one positive PCR test result who were registered in the NAP database, since registration was an entry point to care and treatment under the UC Program. We calculated the proportion of infants with at least one positive PCR test who received a CD4 count and initiated ART within six months and one year of age as of December 30, 2012 (i.e. approximately 15 months after last birth in the study cohort). Lapse time in ART initiation was calculated for each PCR-positive child by subtracting their age (in months) at last PCR test result from their age (in months) at ART initiation.

### Analysis

Infant age at first and second PCR testing was calculated using the date of birth and dates of blood collection. Because actual birth dates are kept confidential by the NHSO and only month and year of birth are recorded, calculations are based on the 15th of the month. In order to adjust the national MTCT rates to include infants who did not access EID services, we did a sensitivity analysis using three scenarios.

In the best-case scenario, we assumed that the MTCT rates in the EID and non-EID groups were the same, as such the MTCT rate was equal to the PCR test positive rate. In a second scenario, we used an estimated weighted average analysis; the proportion of women receiving HAART, dual ARV, single ARV or no ARV was multiplied by the MTCT rate determined in a national survey conducted in Thailand in 2008 (2.0% MTCT rate with HAART, 3.9% with dual ARVs, 9.5% with single ARV [11] and 12.0% with no maternal ARVs). Due to the unavailability of data about women who received HAART from 2009 to 2011, we estimated the proportion of women receiving HAART, dual, single or no ARV using MTCT rates from surveys (2008) [11] and MOPH reporting system (2012) [17] using the interpolating function in Microsoft Excel 2007 (in which the proportion of women receiving HAART ranged from 29% in 2008 to 66% in 2011).

In the worst-case scenario, we assumed that non-EID infants had a higher risk of MTCT as a result of suboptimal PMTCT interventions [18] and used MTCT rates from prior Thai survey data. We assumed 5% of infants received no interventions and had a MTCT rate of 37% [18], 80% received partial/single or dual ARV and had a 9.5% MTCT rate [11], and 15% received triple ARV with a 2.0% MTCT rate [11]. The average MTCT rate of 9.8% in the worst-case scenario was applied to the non-EID infants across the study period to estimate the number of HIV-infections in this group. The Mann–Whitney test was used to test differences in median age at diagnosis, and chi-squared test for linear trend was used to analyze differences in trends of MTCT rates and the other time trend indicators. The Jonckheere–Terpstra test was used to compare the median lapse time of ART initiation from 2008 to 2011.

### Ethical considerations

This study protocol was reviewed and approved by the Ethical Review Committee of the Department of Disease Control, Thailand Ministry of Public Health, and the U.S. Centers for Disease Control and Prevention, Atlanta, Georgia, USA. Because data for this analysis were abstracted by the NAP from routinely collected health information and no individual identifiers were included, the Thai ethical committee waived the requirement for the need for individual informed consent.

## Results

### EID accessibility in health facilities and scale up of sample collection

The number of hospitals collecting EID samples increased from 458 in 2008 to 645 in 2011. Ninety-seven percent of EID samples were collected at public hospitals, with a majority of those (54%) collected in tertiary care hospitals. Community hospitals submitting EID samples increased from 75% (398) in 2008 to 78% (506) in 2011 (*p*=0.044), while the submissions from tertiary care public hospitals and private hospitals remained the same (*p*>0.05). During this same period, the number of infants who received EID in community hospitals increased from 1200 (38%) in 2008 to 1691 (45%) in 2011 (*p*<0.001) (Table 1). The hospitals sending only DBS samples for EID increased from 19% (*n*=349) in 2008 to 37% (*n*=239) in 2011 (*p*<0.001), while the hospitals sending WB samples decreased from 77% (349) in 2008 to 59% (376) in 2011 (*p*<0.001) and both WB and DBS samples remained consistent (*p*=0.568), (Figure 1a). Hospitals providing EID services were distributed throughout the country, with an increasing number of hospitals in remote areas distant from the regional PCR laboratories providing EID services in 2011. This increase corresponded with an increase in the number of hospitals using DBS samples (Figure 1b and c).

### EID uptake, coverage and age of diagnosis

**Figure 2:**
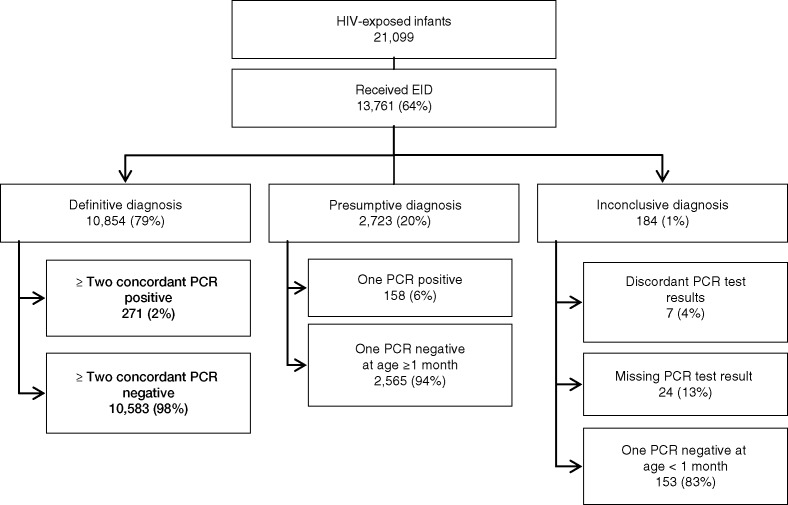
Early infant diagnosis (EID) uptake in the Thai National Program and PCR test results between 2008 and 2011.

From 2008 to 2011, approximately 3.2 million infants were born alive in Thailand, of which an estimated 21,099 infants (0.67%) were born to HIV-positive mothers. EID uptake increased from 3179 in 2008 to 3772 in 2011. The EID coverage increased from 54% (95% CI: 52 to 56%) to 76% (95% CI: 74 to 79%) over this four-year period (Table 1). Of the 13,761 infants who received EID, 10,854 (79%) had at least two PCR tests done and were able to receive a definitive HIV diagnosis. Of those receiving EID, 2723 (20%) had a presumptive diagnosis and 184 (1%) had an inconclusive HIV diagnosis. Of 13,577 infants with definitive or presumptive diagnosis, 429 (3.2%) were PCR test positive (Figure 2). The age of infants at EID sample collection for the first and second tests in 2008 was not different from 2011 (from 2.3 to 2.0 months (*p*=0.08)) for the first sample collection and 4.5 to 4.4 months for the second (*p*=0.08) (Table 2). When stratified by diagnosis, however, the age at second sample collection among the definitive HIV-infected group decreased from 4.6 to 4.0 months from 2008 to 2011 (*p*=0.004), with no change among the definitive HIV-negative group (*p*=0.08) (Table 2). On average, only 40% received the first PCR test before two months of age. The majority (94%) of infants received the first PCR test before they were four months old and 62% received a second PCR test before they were six months old. The proportion of infants receiving their first PCR test before two months of age increased from 35% (1118) in 2008 to 51% (1904) in 2011 (*p*<0.001) (Table 2).

**Table 1:** Number of hospitals, provinces submitting early infant HIV diagnosis (EID), number of live births, HIV prevalence in women giving births estimation of HIV-exposed infants, EID uptake and coverage, 2008 to 2011

	Total	2008	2009	2010	2011	*p*^a^
Provinces submitting samples for EID/total number of provinces, *n* (%)		73/76 (96)	75/76 (97)	75/76 (97)	77/77^b^ (100)	0.105
**Number of hospitals submitting samples for EID**		**458**	**533**	**567**	**645**	
Tertiary care – public (regional, provincial, university) hospitals, *n* (%)		110 (24.0)	116 (21.8)	117 (20.6)	125 (19.4)	0.056
Secondary – primary care (community) hospitals, *n* (%)		337 (73.6)	398 (74.7)	434 (76.5)	506 (78.4)	0.044
Private hospitals, *n* (%)		7 (1.5)	16 (3.0)	14 (2.5)	13 (2.0)	0.923
Unspecified, *n* (%)		4 (0.9)	3 (0.6)	2 (0.4)	1 (0.2)	0.051
**Number of infants received PCR test in different hospitals type**	**13,761**	**3179**	**3444**	**3366**	**3772**	
Tertiary care – public, *n* (%)	7476 (54.3)	1821 (57.3)	1930 (56.0)	1760 (52.3)	1965 (52.1)	<0.001
Secondary – primary care public, *n* (%)	5881 (42.7)	1200 (37.7)	1442 (41.9)	1548 (46.0)	1691 (44.8)	<0.001
Private, *n* (%)	207 (1.5)	26 (0.8)	58 (1.7)	47 (1.4)	76 (2.0)	<0.001
Unspecified, *n* (%)	197 (1.4)	132 (4.2)	14 (0.4)	11 (0.3)	40 (1.1)	<0.001
**Number of registered live births with Ministry of Interior**	**3,147,569**	**797,356**	**787,739**	**766,370**	**796,104**	
HIV prevalence in women giving birth (%)^c^	0.67	0.74	0.69	0.63	0.62	
Estimated number of HIV-exposed infants born	21,099	5900	5435	4828	4936	
EID uptake by HIV-exposed infants	13,761	3179	3444	3366	3772	
EID coverage, % (95% CI)	65 (64 to 66)	54 (52 to 56)	63 (61 to 66)	70 (67 to 72)	76 (74 to 79)	<0.001

a Chi-squared for linear time trend during 2008 to 2011;

b additional newly established province in 2011;

c HIV prevalence data from the national programme for prevention of mother-to-child HIV transmission monitoring system, Department of Health.

**Table 2:** Number and age of infants and hospital type where infants received early infant diagnosis of HIV between 2008 and 2011

	Total	2008	2009	2010^a^	2011	*p* b
Number of infants with ≥1 PCR test positive	429	119	111	109	90	
Number of HIV-infected estimated by						
average scenario (best- and worst-case scenarios)	804	272	212	166	154	
	(683 to 1151)	(226 to 387)	(178 to 307)	(159 to 253)	(119 to 205)	
HIV-infected infants identified by EID programme^c^ (%)						
average scenario (best- and worst-case scenarios)	53 (63 to 37)	44 (53 to 31)	52 (62 to 36)	66 (69 to 43)	58 (76 to 44)	0.316 (0.042, 0.013)
**Median age in months (IQR)**						
Age at first blood collection		2.3 (1.8, 3.3)	2.3 (1.8, 4.1)	2.2 (1.8, 2.8)	2.0 (1.4, 2.5)	0.08
Age at second blood collection		4.5 (4.0, 5.7)	4.4 (3.9, 5.2)	4.4 (3.9, 5.2)	4.4 (4.0, 4.9)	0.077
Definitive HIV-negative diagnosis		4.5 (4.0, 5.7)	4.4 (4.0, 5.2)	4.4 (4.0, 5.2)	4.4 (4.0, 4.9)	0.077
Definitive HIV-positive diagnosis		4.6 (4.0, 6.6)	4.4 (3.6, 6.0)	4.4 (3.6, 6.0)	4.0 (3.1, 4.0)	0.004
**Age of first PCR test**, ***n*** **(%)**						
≤ 2 months	5497 (39.9)	1118 (35.2)	1201 (34.9)	1274 (37.8)	1904 (50.5)	<0.001
> 2 to 4 months	7459 (54.2)	1806 (56.8)	2016 (58.5)	1908 (56.7)	1729 (45.8)	<0.001
> 4 months	791 (5.7)	248 (7.8)	225 (6.5)	182 (5.4)	136 (3.6)	<0.001
Unspecified age	14 (0.1)	7 (0.2)	2 (0.1)	2 (0.1)	3 (0.1)	0.079
**Age of second PCR test**, ***n*** **(%)**						
≤ 4 months	2888 (21.0)	288 (20.0)	744 (21.6)	723 (21.5)	785 (20.8)	0.524
> 4 to 6 months	5644 (41.2)	1138 (35.8)	1405 (40.8)	1402 (41.7)	1719 (45.6)	<0.001
> 6 months	1672 (12.2)	719 (22.6)	627 (18.2)	533 (15.8)	473 (12.5)	<0.001
No second PCR	2857 (20.8)	686 (21.6)	668 (19.4)	708 (21.1)	795 (21.1)	0.901

a Year started WHO Option B regimen for prevention of mother-to-child transmission of HIV, shaded columns are period of Option B implementation;

b chi-squared for linear time trend during 2008 to 2011;

c HIV-infected infants identified by EID programme calculated from infants with PCR test positive at least one time divided by estimated number of HIV-infected infants in each scenario.

### PMTCT programme effectiveness

**Figure 3:**
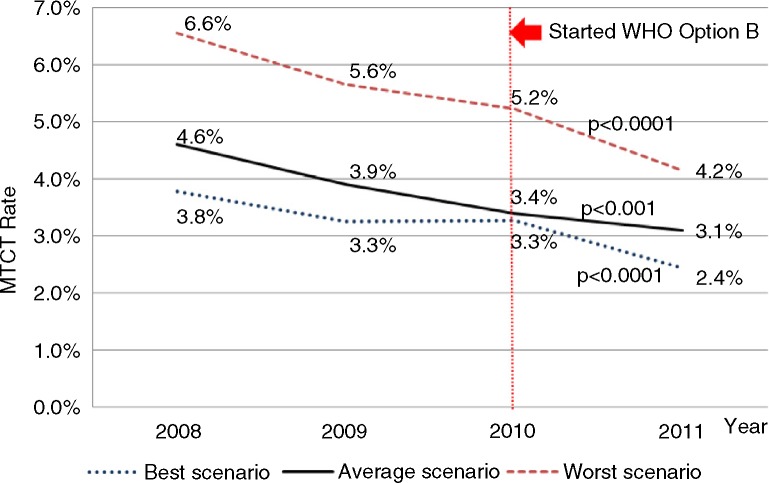
Mother-to-child transmission rates by year of birth adjusted by three scenarios. Chi-squared test for linear trends of all estimated MTCT rates have *p*<0.001.

The MTCT rate in the best-case scenario decreased over time, from 3.8% (226/5900) (95% CI: 3.1 to 4.5%) in 2008 to 2.4% (119/4936) (95% CI: 2.0 to 3.0%) in 2011. The trends in estimated MTCT rates using the weighted average and worst-case scenarios also decreased, from 4.6% (272/5900) (95% CI: 4.0 to 5.2%) in 2008 to 3.1% (154/4936) (95% CI: 2.7 to 3.6%) in 2011 and from 6.6% (387/5900) (95% CI: 5.9 to 7.3%) in 2008 to 4.2% (205/4936) (95% CI: 3.6 to 4.8%) in 2011, respectively. After the introduction of the Option B policy in 2010, MTCT rates continued to decrease (Figure 3).

### Linkage to and continuity of HIV care

From 2008 to 2011, among an estimated 21,099 infants born to HIV-positive mothers, the estimated number of HIV-infected infants was 683 (3.2%), 804 (3.8%) and 1151 (5.6%) using the best-case, weighted average and worst-case scenarios, respectively. Therefore, over the four-year period, the EID programme identified 63% (429/638), 53% (429/804) and 37% (429/1151) of estimated HIV-infected infants by best, weighted average and worst-case scenarios, respectively. There were increasing proportions of HIV-infected infants identified by the EID programme in best- and worst-case scenarios [(53% in 2008 to 76% in 2011, *p*=0.042 and 31% in 2008 to 44% in 2011, *p*=0.013, respectively] but no change in the average scenario (*p*=0.316) (Table 2).

**Figure 4:**
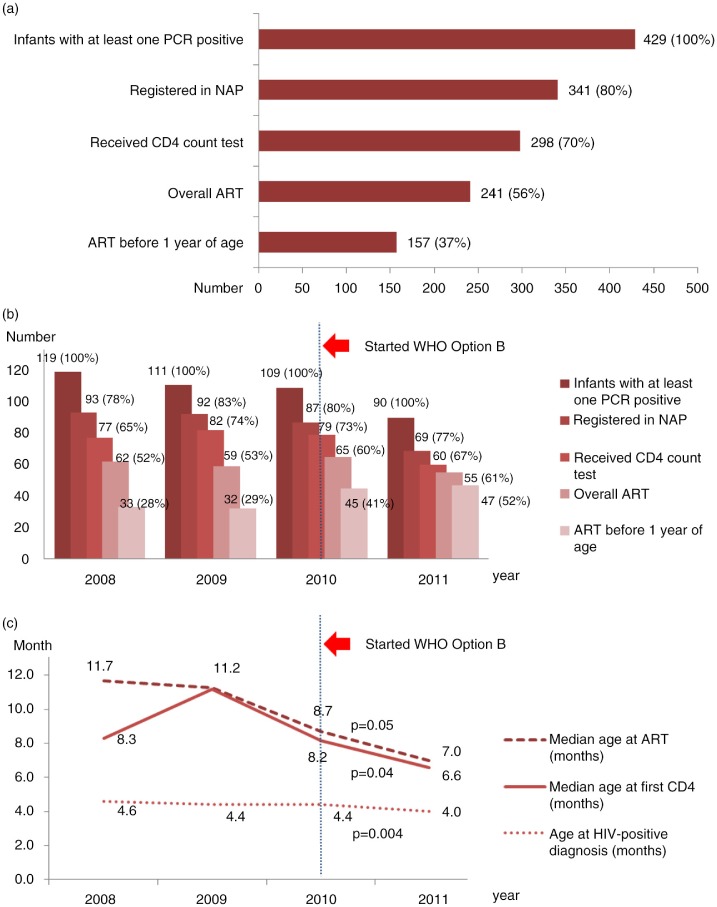
Cascade of PCR-positive infants and linkage to care. (a) Overall cascade of EID and linkage to care, (b) cascade of EID and linkage to care by year of birth and (c) median age of ART initiation, first CD4 count test and at HIV-positive diagnosis significantly decreased by year of birth.

Of 429 infants with at least one PCR-positive test result, 341 (80%) were registered in NAP, and 241 (56%) initiated on ART at any age by the time of data analysis (Figure 4a). Of infants with at least one PCR positive, the proportion initiating ART before one year of age increased from 28% (33/119) in 2008 to 52% (47/90) in 2011 (*p*<0.001) (Figure 4b). There was a decrease in the median age at ART initiation (from 11.7 months in 2008 to 7.0 months in 2011, *p*=0.05) and the age at first CD4 count testing (from 8.3 months in 2008 to 6.6 months in 2011, *p*=0.04). The median lapse time in ART initiation also decreased from 7.2 to 3.8 months (*p*<0.001) during the period of analysis (Figure 4c).

## Discussion

This study analyzed data from a well-established national programme database that can link HIV-exposed and infected infants throughout the whole HIV care cascade and demonstrated that the EID programme in Thailand increased uptake and coverage (54 to 76%) between 2008 and 2011. The availability of DBS sample collection has improved access to EID, demonstrated by the increasing number of hospitals, especially community hospitals, using DBS. The MTCT rate in Thailand has declined to approximately 2 to 3% in recent years, positioning Thailand to achieve, in the near future, the World Health Organization's goal of MTCT elimination. However, the programme should prioritize EID service uptake, and strengthening of the HIV treatment and care cascade, among HIV-infected infants. Early initiation of ART in infants is important to reduce morbidity and mortality [2] and may also prevent the establishment of HIV and integration of HIV DNA in reservoir sites [19], which may allow a subsequent ART interruption period, thus preserving future treatment options [20]. This study found that the age at first PCR test was older than that recommended in national guidelines, and rates of ART initiation in HIV-infected infants within one year of age were relatively low. EID services should be expanded to cover all HIV-exposed infants, including two PCR tests for definitive diagnosis, in accordance with national guidelines. Poor compliance with infant ART guidelines may be due to its complexity, the delayed rollout of the guidelines, lack of healthcare provider's confidence in starting ART in young infants [21], infants being lost to follow-up or having died before HIV diagnosis [22] and limited availability of paediatric ARV formulations [23]. In addition, delays in the EID process, including the return of HIV test results to caretakers [24] and the requirement for a paediatrician to initiate therapy, may also contribute to late ART initiation [21]. However, low ART initiation rates before 2011 may be also due to the ART initiation criteria in national guidelines at the time, while improvement was observed after the revision of the guidelines [25]. PCR testing at birth may play an important role in detecting *in utero* HIV infection, reducing loss to follow-up, improving early ART and saving infants from AIDS-related death [26].

In response to these study findings, the Thai MOPH has established an active follow-up and management system for HIV-positive pregnant women and their exposed infants, to improve EID programme coverage, linkage to care and early ART initiation in HIV-infected infants. Training in case management for early identification and infant ART initiation was conducted throughout the country in 2013 to 2014. The revised national guidelines in 2014 recommend earlier PCR testing: at birth (where feasible), at one month and at two to four months, and recommend ART initiation as soon as possible if PCR-positive infants are identified [5]. These changes are expected to improve EID coverage, lead to earlier ART initiation and reduce the leakage of HIV-infected infants from the HIV treatment cascade.

In order to validate progress towards MTCT elimination and evaluate programme effectiveness, reliable estimates of the MTCT rate must be made. The number of positive PCR test results over a specified time period can provide a good estimate of the MTCT rate, especially with the increasing coverage of PCR testing in a predominantly non-breastfeeding population like Thailand. However, MTCT rates estimated using PCR test results may underestimate the true MTCT rate. Children who did not receive a PCR test were more likely to have mothers who received suboptimal ANC and ARV regimens than those who did have a PCR test; thus, in these individuals, a higher risk of MTCT could be expected [18]. In order to account for unknown factors, this study used sensitivity analysis with adjusted scenarios to determine best- and worst-case scenarios of MTCT rates in Thailand.

### Strengths and limitations

Using population-based routine programme data that are mandatory for healthcare cost reimbursement allows longitudinal follow-up of HIV-exposed infants from EID access through the continuum of HIV diagnosis, treatment and care. Data from this system cover the majority of HIV-exposed infants born, availing a robust data set for evaluating national PMTCT programme effectiveness.

The results of this study are subject to several limitations. These data were obtained from a database that was designed for routine HIV programme management; therefore, it is possible that human error occurred during data entry. We may have underestimated the number of HIV-exposed infants because an estimated 5% of infants born each year in Thailand are not registered with the national birth registration system [27]. The EID coverage rate may be underestimated since non-Thai infants can be registered with the national birth registration system and access EID services through special programmes that were not included in the NAP database. Using interpolation data for ARV used by HIV-positive pregnant women may have overestimated MTCT rates if the increase in HAART uptake was not linear. A separate Thai MOPH survey reported that the uptake of HAART regimens by HIV-positive pregnant women were 27% in 2008 and 73% in 2011 (unpublished data), while the interpolation estimates were 29% in 2008 and 66% in 2011. To avoid the possibility of a false PCR negative result before one month of age, we excluded infants who had only one negative PCR test result before one month of age. This may have overestimated the MTCT rates in PCR-tested infants. The adjusted MTCT rates in the weighted average and worst-case scenarios may be overestimated due to use of MTCT rates from prior studies when HAART prescription was based on CD4 rates less than 200 cell/µl. Transmission rates during that period may have been higher in these immunocompromised mothers. Infants lost to follow up may have died from HIV-related causes that could not be captured in this database. This may have led to an underestimated MTCT rate. Finally, this study did not include HIV incidence in pregnant women in the MTCT rate estimation because of low HIV seroconversion rates during pregnancy found in an earlier study [28]. Repeated HIV testing during the third trimester is routinely done in Thailand.

## Conclusions

This study found an increase in national EID programme uptake and coverage over time, improvement in national PMTCT programme effectiveness as evidenced by decreasing MTCT rates and a high proportion of overall linkage to HIV care among infants with positive PCR test results. There remain, however, a substantial number of infants with a positive HIV diagnosis who were not initiated on ART within one year of age. In response to these findings, the Thai MOPH is implementing an active case management programme to more effectively identify infants at risk of HIV exposure, provide EID services and improve linkages to care and early ART towards the goal of MTCT elimination.
